# The French Armed Forces Virology Unit: A Chronological Record of Ongoing Research on Orthopoxvirus

**DOI:** 10.3390/v10010003

**Published:** 2017-12-23

**Authors:** Déborah Delaune, Frédéric Iseni, Audrey Ferrier-Rembert, Christophe N. Peyrefitte, Olivier Ferraris

**Affiliations:** Unité de virologie, Centre National de Référence-Laboratoire Expert Orthopoxvirus, Institut de Recherche Biomédicale des Armées, 91220 Brétigny-sur-Orge, France; deborah.delaune@intradef.gouv.fr (D.D.); frederic.iseni@intradef.gouv.fr (F.I.); audrey.ferrier-rembert@intradef.gouv.fr (A.F.-R.); christophe.peyrefitte@intradef.gouv.fr (C.N.P.)

**Keywords:** orthopoxvirus, smallpox, bioterrorism, emergence, vaccines, Lister strain, antivirals, genome replication

## Abstract

Since the official declaration of smallpox eradication in 1980, the general population vaccination has ceased worldwide. Therefore, people under 40 year old are generally not vaccinated against smallpox and have no cross protection against orthopoxvirus infections. This naïve population may be exposed to natural or intentional orthopoxvirus emergences. The virology unit of the Institut de Recherche Biomédicale des Armées (France) has developed research programs on orthopoxviruses since 2000. Its missions were conceived to improve the diagnosis capabilities, to foster vaccine development, and to develop antivirals targeting specific viral proteins. The role of the virology unit was asserted in 2012 when the responsibility of the National Reference Center for the Orthopoxviruses was given to the unit. This article presents the evolution of the unit activity since 2000, and the past and current research focusing on orthopoxviruses.

## 1. Introduction

After decimating millions of people during several centuries, smallpox was finally eradicated in 1980, due to the worldwide massive vaccination campaigns organized by the World Health Organization (WHO) in the 1960s and 1970s [1]. By 1984, the vaccination in the general population was stopped because of unfavorable benefit–risk balance caused by vaccination complications. All strains of variola virus (VARV) and clinical samples were destroyed, except in the two repositories that were authorized by the WHO. Even if the destruction of VARV residual stocks was frequently questioned, orthopoxviruses had never disappeared from public health issues. Bioterrorist attacks in September 2001 called back the possible use of VARV as a biological weapon. Moreover, outbreaks of monkeypox virus (MPXV) in the United States in 2003 [2], more recently in Africa [3], and recent vaccinia virus (VACV) outbreaks in Brazil [4], highlight the natural risk due to the emergence of pathogenic orthopoxviruses in vaccine-naïve populations. Considering the natural outbreaks and the potential intentional use of orthopoxvirus warfare agents, numerous preparedness plans were set up. In France, the facility that currently promotes the French military biomedical research was designated in 2012. The Institut de Recherche Biomédicale des Armées (IRBA, Brétigny sur Orge) inherited the research programs from CRSSA (Centre de Recherche du Service de Santé des Armées) located in Grenoble, IMTSSA (Institut de Médecine Tropicale du SSA) located in Marseille, and IMNSSA (Institut de Médecine Navale du SSA) located in Toulon. The major research programs encompassed the development of medical countermeasures against chemical, biological, radiological, and nuclear (CBRN) agents, and the physiological and cognitive aspects of soldier health under challenging environmental conditions. The virological research programs were hosted by the virology unit located at the IRBA following the closure of the historical military medical research institutes. The CRSSA virology research unit had been asked to start a research program on orthopoxviruses in 2000. The strategy was to enhance the basic knowledge of these viruses, aiming at developing medical countermeasures against VARV using surrogate viruses such as VACV. During the last decade, diagnosis capabilities were built at the national level [5], leading the Ministry of Health to give the unit the responsibility to create and hold the first National Reference Center for the Orthopoxviruses (NRC OPX) in January 2012 (26 December 2011 decree fixing the list of National Reference Centers aiming to fight infectious diseases). The research programs included the development of the complete poxvirus diagnosis capability, the survey of these viruses, the development of antiviral molecules, and vaccine candidates. In 2017, the name NRC OPX was modified into NRC OPX-Expert Laboratory (NRC-EL-OPX) (7 March 2017 decree fixing the list of National Reference Centers and National Reference Centers Expert Laboratories aiming to fight infectious diseases). The NRC-EL-OPX is dedicated to detecting and identifying the orthopoxviruses of medical importance circulating in France mainland and French overseas territories. More fundamental studies focusing on the development of new antiviral strategies and novel vaccine candidate design are also performed in the virology unit, and are summarized in this paper.

## 2. The Poxviruses

**Figure 1:**
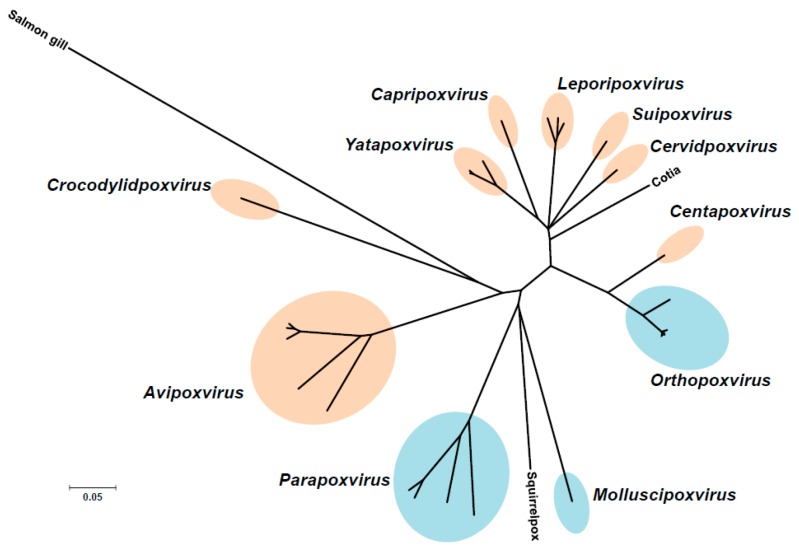
Genetic diversity of poxviruses. Genus in which French National Reference Center-Expert Laboratory for Orthopoxvirus (NRC-EL-OPX) isolates had been identified is highlighted in blue.

Poxviruses are responsible for diseases of medical importance in human beings and animals worldwide. These DNA viruses of the *Poxviridae* family, whose genome length is varies from 135 to 360 kb, can be grouped into two subfamilies: *Entomopoxvirinae* and *Chordopoxvirinae*, infecting insects and vertebrates, respectively. Based on host range, morphology, antigenicity, and sequence similarity, each of these subfamilies is subdivided into genera. The *Chordopoxvirinae* subfamily is composed of 11 genera: *Avipoxvirus, Capripoxvirus, Centapoxvirus, Cervidpoxvirus, Crocodylipoxvirus, Leporipoxvirus, Molluscipoxvirus, Orthopoxvirus, Parapoxvirus, Suipoxviruses, Yatapoxvirus* genus (Figure 1). Members of the subfamily *Chordopoxvirinae* have a broad animal reservoir, mainly related to rodents. Viruses that are members of the *Parapoxvirus, Yatapoxvirus, Orthopoxvirus*, or Molluscipoxvirus genera infect a wide range of mammals, including humans either exclusively (i.e., VARV and molluscum contagiosum virus, MCOV) or both humans and animals. With rare exceptions, most human poxvirus infections, which usually occur through minor skin abrasions, fail to establish a human chain of transmission. A total of 14 poxviruses have been documented to infect humans [6], seven of which belong to the *Orthopoxvirus* genus (VARV, VACV, MPXV, cowpox virus (CPXV), buffalopox virus, cantagalo virus, aracatuba virus), one to the *Molluscipox* genus (MCOV), and one to the *Yatapoxvirus* genus (tanapox virus). The remainder belonged to the *Parapoxvirus* genus (orf virus, pseudocowpox virus (PCPV), bovine papular stomatitis virus, deerpox virus, and sealpox virus) [7]. MPXV, CPXV, orf virus, and MCOV cause the most frequent human poxvirus infections worldwide.

## 3. French National Reference Center-Expert Laboratory for Orthopoxvirus

The NRC-EL-OPX, as a result of its diagnostic activity, is consequently part of the alert system allowing the implementation of the smallpox national response plan in France. Nowadays, with the exception of the population vaccinated against smallpox virus or partially protected after other orthopoxvirus exposition (such as CPXV), people < 40 year old, which had neither been vaccinated against, nor exposed to smallpox virus, remain vulnerable to the virus. To date, the residual protection of the smallpox vaccinated population (i.e., people > 45 year old) is not clear. Indeed, seroprevalence studies have demonstrated high antibody levels in this population that are not always correlated with efficient protection [8,9,10]. The importance of MPXV in human health has also been recognized because of the successive epidemic outbreaks in Africa since 1995, with fatality cases, and its introduction in the United States in 2003 [11]. Orthopoxvirus disease, including smallpox, is a notifiable disease in France, whose epidemiological surveillance is carried out by the NRC-EL-OPX. The missions of the NRC-EL-OPX, defined by the 16 June 2016 decree, are dedicated to control poxvirus communicable diseases and to advise the governmental authorities through the identification and characterization of the pathogens. The NRC-EL-OPX is also part of the Biotox network of laboratories spread across the country to tackle (CBRN) outbreaks.

**Figure 2:**
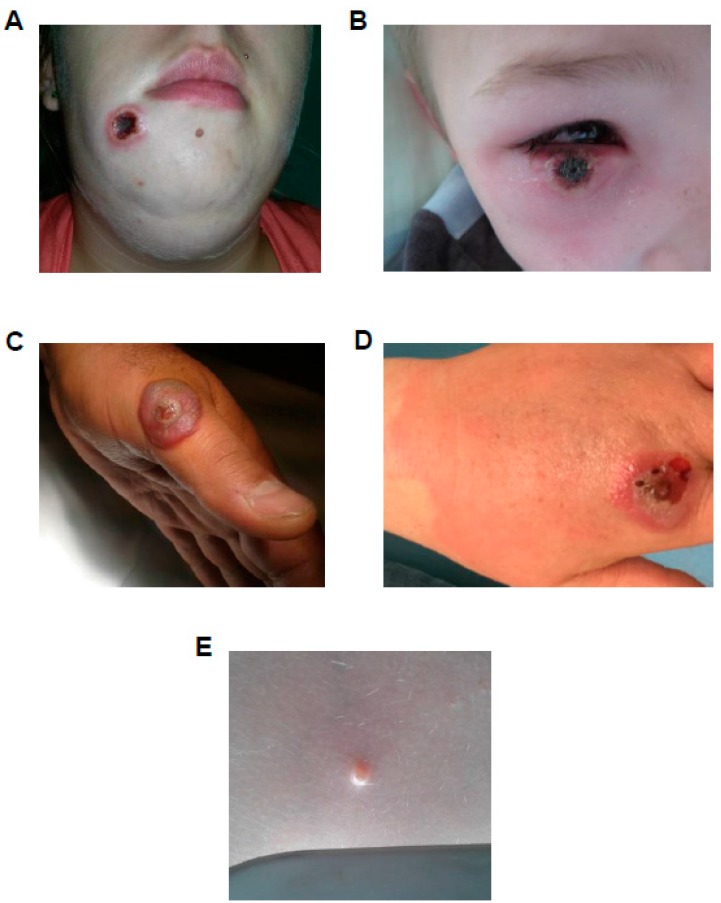
Poxvirus infection cases treated at the NRC-EL-OPX. Patients with lesions due to cowpox virus (CPXV) (**A**,**B**), orf virus (ORFV) (**C**), pseudocowpox virus (PCPV) (**D**) and molluscum contagiosum virus (MCOV) (**E**) infection. Lesions are located on the face (**A**,**B**), on the thumb (**C**), on the hand (**D**) and in the back (**E**).

A confirmed case is defined by at least one of the following criteria: (i) direct detection of the virus in cell culture; (ii) amplification of viral genomic DNA using PCR [5]; (iii) visualization of viral particles by electron microscopy; (iv) detection of viral antigens, and (v) indirect detection of viral antibodies in serological assays. Since 2012, the NRC-EL-OPX was able to identify 8 orthopoxvirus, 24 parapoxvirus, and 5 molluscipoxvirus human cases. Although the orthopoxvirus case number was low, severe clinical presentations were observed [12,13]. CPXV was identified in all of these cases, emphasizing the role of the rodents in the maintenance of the virus [14]. CPXV infections cause skin lesions. They are often epitheliotropic, starting as vesicular lesions, then pustules with an indented center and raised erythematous borders which evolve towards the crust stage (Figure 2A,B). Inflammation can be observed, especially in ocular infection. The infection may be associated with fever and adenopathy, particularly when the lesion is located in the head. The lesions observed in cases reported by the NRC-EL-OPX were isolated and observable at palmar level, the wrists, the head, the cheek, and the eyes [12,13].

Indeed, small wild rodents are believed to constitute the natural reservoir of CPXV, but this virus has a broad host range, and may infect a wide variety of mammals such as cats, horses, elephants, dogs, and humans. However, proven inter-human transmission is due to close contact to a few numbers of these intermediary hosts, such as rats purchased from a pet shop. An outbreak showed the role of the pet rats grown in central Europe [13,15,16]. Close contact with cats that go outdoors are also known to transmit CPXV to humans. In this case, simple contact by skin or a mucosa lesion seems to increase the virus transmission [12]. Although self-inoculations of the virus in different areas, such as eyes, wrists, and hands had been observed, no human-to-human transmission had been reported yet. Out of the eight orthopoxvirus cases diagnosed by the NRC-EL-OPX, six were associated with people aged from 2 to 22 year old.

Out of the 82 suspected cases reported in France during the 2012–2017 survey period, 24 parapoxvirus cases, including 20 orf viruses, were confirmed. Parapoxvirus infections cause skin lesions, papules form, followed by vesicles, and finally, wart-like nodules. An iris-like lesion can develop with red center, white middle ring, and erythematous periphery. In the vast majority of the cases, the lesion is located in the hand (Figure 2C). These cases were mostly associated with direct contact with infected sheep, or contaminated carcasses during the Eid ritual animal slaughters. The remaining four cases were due to the PCPV in cattle producers (Figure 2D). The infection is not restricted to one age group, but is rather related to the proximity of the patient to an intermediate host, such as sheep or cow. Consequently, the majority of cases were associated with adults aged from 31 through 60 year old. Only 3 cases were associated with people aged from 11 through 30 year old. The NRC-EL-OPX reported five MCOV cases: four were from children under 10 year old, and surprisingly one from a 45 year old patient (Figure 2E). MCOV, the lone species in the genus *Molluscipoxvirus*, is a human-specific pathogen which causes serious symptoms only in immunocompromised individuals [17]. It is a common and mild children’s disease that is diagnosed by simple clinical examination. That is the reason why the NRC-EL-OPX received only a small number of biological samples for diagnosis.

## 4. Smallpox Vaccine, Past and Present

Two strains of VACV were used for worldwide mass vaccination: the New York City Board of Health (NYCBH) strain and the Lister strain developed in the city of Elstree (England) at the Lister institute. While the NYCBH vaccine was used mainly in the United States, the original stock of Lister vaccine prepared in 1961 was provided to Paris, Tokyo, Atlanta, and Moscow, to be finally used by 23 of the 59 vaccine producers worldwide. This is the original strain of vaccine with an authorization by the health authorities in most of the European countries, including France (first produced by Pourquier vaccine, and then Sanofi-Pasteur) [18]. These first generation vaccines had limitations because of adverse effects (eczema vaccinatum, vaccinia necrosum, etc.) [19,20] and contraindications (immunodeficiencies, pregnancy, skin lesions, etc.) [21]. A major concern regarding these vaccines was their production on the flank of cows or sheep, from which the vaccine pulp was then extracted by scraping, and grossly purified by centrifugation [22]. The final product was stocked as a liquid or freeze-dried at an infectious titer of 10^8^ pfu/mL [18]. The vaccine product was not sterile, and the bacteria had to stay under 500 germs/mL to respect the WHO requirement.

In France, this vaccine was inoculated by scarification in the deltoid. Ten microliters of vaccine were administered using two vaccinostyle scarifications containing a drop of 10^6^ pfu vaccine dose, followed by a recommended boost [23].

**Figure 3:**
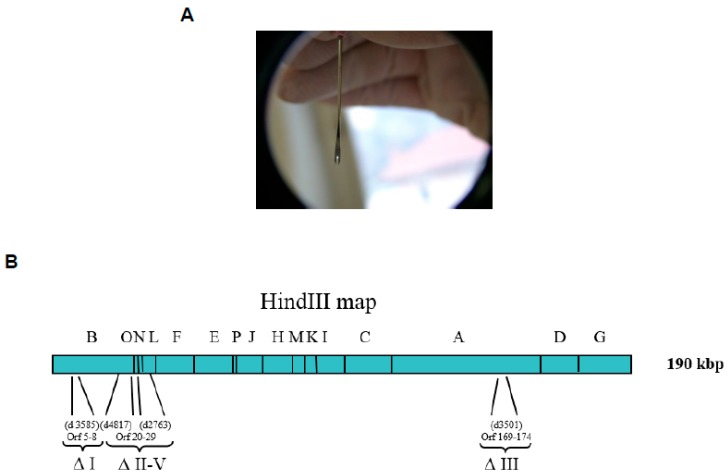
Bifurcated needle (**A**). Modified Vaccinia Lister (MVL) genome scheme with localization of deletions within the vaccinia virus (VACV) Lister-107 genome and the positions of the HindIII restriction fragments (**B**). The numbers in parentheses below the viral genome indicate the approximate locations of MVL deletions previously mapped in the Modified Vaccinia Ankara (MVA) strain relative to the Copenhagen strain (from left to right, according to the nomenclature of Antoine et al. [31]). The actual open reading frames (ORFs) that were deleted in the VACV Lister genome are identified beneath the indicated MVA deletions, and numbered according to Garcel et al. [29]. The roman numeral nomenclature shown under the deleted genomic regions described in Meyer et al. [32].

After smallpox eradication, the Lister vaccine production was stopped. Following the 2001 terrorist attacks, the French national authorities have decided to requisition all the remaining stocks (4.8 million doses of Pourquier vaccine, of which 500,000 are military doses) [24] to face a potential smallpox outbreak. However, in addition to well-known contraindications and adverse effects, French national health authorities were confronted with the insufficient number of vaccine doses in case of necessity. Thus, the first challenge was to increase the number of vaccine doses. The virology unit, then at the CRSSA, showed in non-human primate (NHP) models that the use of bifurcated needles (Figure 3A) could significantly reduce the amount of vaccine required to inoculate [18,24]. Of note, the bifurcated needle vaccination consists of 0.9 µL of VACV vaccine injected with 15 multi-punctures, compared to the previous 10 µL vaccinostyle VACV vaccination. As a consequence, this administration mode allowed a ten times increase in vaccine dose availability for the total French population [18,24]. This advance in smallpox vaccination has been reinforced by the production of 21.5 million doses of first generation vaccine by Sanofi-Pasteur in 2002, increasing the number of available doses to 95.4 million [24].

Later on, the development of new vaccines appeared to be a necessity, because of the numerous persons that could potentially be excluded from the first generation Lister vaccination. Again, the virology unit took part to this development in which preclinical studies had shown the efficacy of a second generation smallpox vaccine (same strain of first generation vaccine, but produced in cell culture) from Sanofi-Pasteur VACV [25,26]. Our work had also shown the lack of efficacy of the third generation smallpox vaccine candidate, the highly attenuated Copenhagen vaccinia virus (VACV) derived NYVAC strain [27].

A third generation non-replicative smallpox vaccine (MVA, modified Vaccinia Ankara) attenuated in cell culture could be used in mass vaccination, including persons with contraindication to the first generation vaccine. However, this safe vaccine requires high intramuscular dose vaccination, and no long-term protection was observed in mice models [28]. In parallel, the virology unit had developed its own third generation smallpox vaccine candidate as follows. The first generation Pourquier Lister strain vaccine was cloned, and the most representative clone was isolated as VACV-107 [29]. Targeted deletions of the genome were designed based on MVA to trigger attenuations (Figure 3B). Recombinant viruses harboring different deletion combinations were analyzed according to their cell culture attenuation, safety using NUDE and SCID mice, protection against a CPXV lethal challenge, and the induction of the cell- and humoral-specific immune responses [30] (European patent number EP08305570.7, U.S. patent numberUS2011017125611). The best mutant was named MVL (Modified Vaccinia Lister) [30]. Although this vaccine candidate was less replicative than the first generation, it did not induce any mortality nor morbidity in SCID and NUDE mice [30]. Moreover, comparable cellular and humoral immune responses were observed when 10^8^ pfu MVA was intramuscularly injected, in comparison to a 10^5^ pfu MVL scarification.

Finally, like other VACV, MVL is an efficient vector for exogenous gene expression. In the virology unit, three bivalent vaccine candidates were designed against hemorrhagic fever viruses, such as Zaire Ebola virus (MVL-ZEBOV), Crimean Congo Hemorrhagic Fever (MVL-CCHFV) and Rift Valley Fever (MVL-RVFV). Evaluation of the effective protection of these vaccine candidates in animal models is in development.

## 5. Antivirals

The developments of preventive and curative antiviral molecules are complementary approaches to control orthopoxvirus infections. These antivirals will be useful to protect and cure not only exposed and infected persons, but also patients whose vaccination is contraindicated or leads to complications. Antiviral drugs against VARV prove difficult to develop, since most research occurred after smallpox eradication, thus preventing clinical trial feasibility. Currently, there is no US Food and Drugs Administration (FDA) approved molecule for the treatment of orthopoxvirus infection. However, cidofovir (CDV) and ST-246 can be exceptionally used to control orthopoxvirus infection.

CDV (Vistide^®^), an acyclic nucleoside analog of deoxycytidine targeting the poxvirus DNA polymerase, was first described in 1986 [33]. It presents a broad antiviral spectrum including poxviruses, herpesviruses, or adenoviruses [34]. In 1996, it was approved for the treatment of cytomegalovirus retinitis in AIDS patients. CDV inhibits viral genome synthesis acting as a chain terminator, and its incorporation in a synthesized DNA strand interferes with the DNA polymerase 3′–5′ exonuclease activity [35]. It was the first Center for Disease Control and Prevention (CDC) recommended drug to treat smallpox, in case of an intentional VARV release [36]. CDV was included in strategic stockpiles in several countries. However, its synthesis was stopped in 2013, because of industrial production issues. The major limits of this compound were its intravenous administration and its high nephrotoxicity. In order to improve the CDV biodisponibility, a lipidic ester known as CMX001 (Brincidofovir) was synthesized. Brincidofovir was shown to inhibit VACV and VARV replication in vitro [37,38]. Brincidofovir efficacy in vivo was demonstrated in mousepox virus infected mice [39], and in rabbitpox virus infected rabbits [40]. The main advantages of Brincidofovir are the oral administration and the absence of nephrotoxicity [41]. This drug had a positive safety profile for smallpox treatment [42,43]. In October 2016, the European Drug Agency categorized CMX001 into the orphan status for smallpox treatment. Moreover, Brincidofovir is under the US FDA’s Animal use, and an FDA approval has been requested. A response is expected in 2018.

ST-246 was identified via a high-throughput screening using VACV infected Vero cells [44]. It inhibited VACV, CPXV, and camelpox virus in vitro [45], along with VARV and MPXV [46]. Its efficacy was also demonstrated in a murine model [47], and in VARV-infected cynomolgus monkey [48]. ST-246 inhibits virion egress by interacting with the viral protein F13. It has a good oral bioavailability and is stable at room temperature. Phase 1 clinical trials have been realized [49]. The proposed smallpox treatment regimen is 400 mg or 600 mg per day for 14 days [50]. ST-246 was successfully used in association with vaccinia virus immune globulin and CDV [51] in rare cases of eczema vaccinatum or vaccination complications [52]. The FDA approval is expected in 2018.

Since 2005, the virology unit has developed studies aiming at deciphering the atomic structure of essential proteins necessary for poxvirus genomic DNA synthesis. These projects will not only help understanding the molecular mechanisms involved in the viral replication, but also, will be useful for the future development of compounds targeting these key proteins. Our work focused, in particular, on VACV E9, D4, A20, and D5, four proteins present at the replication fork. E9 is the catalytic subunit of the DNA polymerase [53], and the target of CDV and other antipoxvirus compounds [54]. D4 is an uracil-DNA glycosylase [55,56] that interacts with A20, forming the heterodimeric E9 processivity factor [57]. D5 is a hexameric nucleoside triphosphatase [58,59] which contains a superfamily III helicase domain [59,60] and shows primase activity [61].

**Figure 4:**
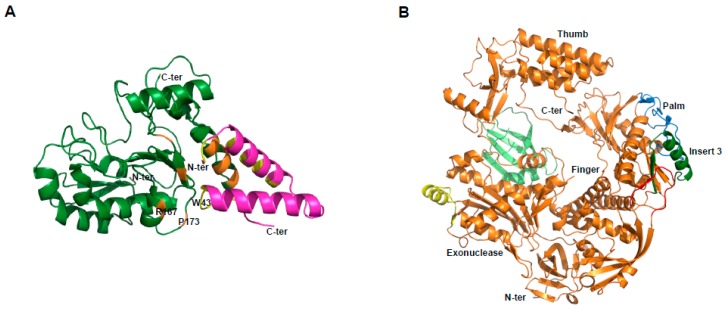
Crystallographic structures of D4/A20_1-50_ and the DNA polymerase of VACV. (**A**) D4 is depicted in dark green, and A20_1-50_ in magenta. In orange and yellow are shown the residues of D4 and A20, respectively, critical for the D4/A20 contact. Residues R167, P173 of D4, and W43 of A20 are indicated; (**B**) view of the domain organization. The classical palm, thumb, finger, and exonuclease domains of family B polymerase are shown. The various poxvirus-specific structural insertions are depicted in color. Some residues in insert 3 (dark green) were shown to be critical for E9/A20 interaction [71].

We first established the expression of these proteins in the baculovirus-insect cell system. E9 and D5 were expressed alone, while A20/D4 was expressed as a complex [62]. Electron microscopy studies revealed that D5 forms a hexameric structure, in agreement with its previous classification as a member of the SF3 family [63]. Using small-angle X-ray scattering (SAXS) experiments, we were able to show that the VACV DNA polymerase holoenzyme formed by E9 bound to its processivity factor A20/D4 is found in a 1:1:1 stoichiometry. It was further demonstrated that E9 interacts with A20, but not with D4, and a distance of 150 Å was calculated between the active sites of E9 and D4 that could be linked by 50 to 60 bp of double-helical DNA [62]. In order to obtain greater details of the holoenzyme architecture, high-resolution structures were necessary. A first crystallographic study of D4 bound to the first 50 amino acids of A20 (D4/A20_1-50_) revealed that the A20/D4 heterodimeric complex is formed by a large hydrophobic contact area, and a remarkable feature where Trp43 of A20 is inserted between Pro173 and Arg167 of D4 [64]. As it was previously demonstrated for Leu7, Leu10, and Leu14 [65], mutant studies showed the critical role of Trp43 and Pro173 in complex formation [66] (Figure 4A). A following study also showed in great detail how D4 interacts with a 10-mer DNA duplex containing an abasic site resulting from the cleavage of a uracil base [67]. Since the binding of D4 and A20 is essential for processive polymerase activity, we and others believed that disrupting this interaction with small molecules could represent an interesting strategy for poxvirus inhibition [68,69,70]. The atomic structure of the A20/D4 interface will help future research to achieve this goal.

More recently, we were successful in solving the crystal structure of the catalytic subunit of the VACV DNA polymerase ([71], Figure 4B). The structure allowed us to delineate E9-specific inserts, and to position the enzyme in a global model of the DNA polymerase holoenzyme. We showed that an insertion within the palm domain binds to A20, and represents a mode of processivity factor binding that differs from other members of family B polymerases [71]. As mentioned above, E9 is the target of CDV and its derivative CMX001, and also other inhibitors, such as aphidicolin, phosphonoacetic acid, and cytosine arabinoside. The high-resolution structure of the polymerase active site will accelerate the development of new antiviral drugs. Furthermore, the identification of the E9/A20 interface will facilitate the design of new compounds or peptides that disrupt this interaction [71].

## 6. Conclusions

Following the smallpox eradication, the major risk of re-emergence of VARV was thought to be the result of a misappropriation of the virus retained in the two authorized repositories, or the reappearance of existing strains still viable in the environment. However, a new risk was seriously considered by the WHO, based on the report of the Independent Advisory Group on Public Health Implications of Synthetic Biology Technology Related to Smallpox [72]. Indeed, the risk of potential recreation of VARV is facilitated by the published viral genomic sequences. Recently, a Canadian team headed by D.H. Evans demonstrated the feasibility of recreating an infectious horsepox virus after de novo synthesis of the viral genome from DNA fragments [73]. This work indicates that even if the residual VARV samples were destroyed, the reemergence of VARV in the population will remain a possibility.

Considering these new risks and the potential emergence of other pathogenic orthopoxviruses in humans, such as MPXV, the WHO recommended the development of two major diagnostic axes i.e., the clinical diagnosis and the point of care tests, together with the antiviral research focusing on the multiviral targets to prevent viral emergence resistance [72]. A broad multidisciplinary preparedness strategy is a necessity, which was considered in our virology unit several years ago, and will be consolidated in the future. Our new objectives are to make clinicians aware of poxvirus disease, and develop a network of labs with poxvirus diagnostic capabilities. The NRC-EL-OPX will pursue biological collections for epidemiological studies and treatment resistance surveys. In parallel, research programs will intend to take advantage of the MVL vaccine platform to develop multivalent vaccine candidates. Finally, structure-based drug design will be employed to identify compounds able to inhibit the function of critical proteins involved in poxvirus DNA replication.
